# Transient enhanced cell division by blocking DNA synthesis in *Escherichia coli*

**DOI:** 10.1099/mic.0.000888

**Published:** 2020-03-02

**Authors:** Carmen Mata Martín, Arieh Zaritsky, Itzhak Fishov, Elena C. Guzmán

**Affiliations:** ^1^​ Departamento de Bioquímica Biología Molecular y Genética, Universidad de Extremadura, Badajoz 06071, Spain; ^2^​ Faculty of Natural Sciences, Ben-Gurion University of the Negev, Beer-Sheva 84105, Israel; ^3^​ Department of Life Sciences, Ben-Gurion University of the Negev, Beer-Sheva 84105, Israel; ^†^​ Present address: CICAB Clinical Research Centre, Extremadura University Hospital and Medical School, Badajoz, Spain

**Keywords:** DNA replication, cell division and dimensions, nucleoid morphology

## Abstract

Duplication of the bacterial nucleoid is necessary for cell division hence specific arrest of DNA replication inhibits divisions culminating in filamentation, nucleoid dispersion and appearance of a-nucleated cells. It is demonstrated here that during the first 10 min however, *Escherichia coli* enhanced residual divisions: the proportion of constricted cells doubled (to 40%), nucleoids contracted and cells remodelled dimensions: length decreased and width increased. The preliminary data provides further support to the existence of temporal and spatial couplings between the nucleoid/replisome and the sacculus/divisome, and is consistent with the idea that bacillary bacteria modulate width during the division process exclusively.

## Introduction

One of the classical modes to inhibit DNA replication in bacteria is by depriving thymine of *thyA* mutants, deprivation that gradually stops divisions and hence is associated with filamentation [1, 2], culminating in loss of colony-forming ability namely thymine-less death [3, 4]. Important events such as aborted initiations were recently identified during the first minutes of treatment [5], triggering us to investigate cell and nucleoid dimensions in an attempt to detect possible changes during the *immediate* period after imposing thymine starvation.

## Methods

Three *Escherichia coli* strains, all in our lab collections were used: K12 MG1693 (*thyA715*, *rph-1*) is a spontaneous thymine-requiring derivate of strain MG1655; K12 CR34 (*thr-1, leuB6, lacY1, supE44, rfbD1, thi-1, mcrA1, cyn-1, deoC1, thyA6, fhuA2*); 15 TAU-bar (*thyA42*, *deoB20*, *ura*, *arg*, *met*, *pro*, *trp* (from PC Hanawalt). A single colony from a nutrient agar (NAT) plate, was inoculated in M9 minimal medium with thymine 20 µg ml^−1^, casaminoacids 0.2 % and glucose 0.4%, and incubated overnight by shaking at 37 °C. This culture was diluted 1 : 200 in fresh medium and maintained in the exponential phase for at least six mass doublings. Growth was monitored by absorbance at 550 nm (OD_550_). Changes in conditions were usually performed at OD_550_ between 0.15–0.2. All materials and tools used were pre-warmed (37 °C). Colony-forming ability (CFA) was determined in duplicate on NAT plates at 37 °C and normalized to time 0. Experiments were repeated at least thrice.

Exponentially growing culture at OD_550_=0.2 was collected on a nitrocellulose Millipore HA WP04700 filter (47 mm diameter; 0.45 µm pore diameter) using a Millipore vacuum pump, allowing a minimal time to reach the new condition. Filtered cells were washed with 5×-volume of the same medium without thymine, pre-warmed, and re-suspended by vortex (1 min) in the same volume of pre-warmed medium without thymine. Plates were incubated under the same conditions. Treatments such as addition of drugs were performed simultaneously.

A 200 µl sample was fixed in 50 µl formaldehyde 1.25 % and stored at room temperature. To visualize the nucleoid, DNA was stained with DAPI at 1 µg ml^−1^.

To immobilize and spread cells in a single focal plane, a smooth 2 % agarose surface was prepared (gelling point 35 °C, SERVA-A1U04, Heidelberg) in growth medium and kept at 60–70 °C. A 100 µl aliquot was poured onto an object slide and covered with a siliconized coverslip (22×50 mm). After solidification, the cover slip was carefully removed resulting in an agarose ‘microslab’. A 4 µl drop of a concentrated cell suspension was added and covered with a coverslip.

Images were acquired by an inverted Olympus microscope (IX70) equipped with a Micromax 512 camera (Princeton Instruments) and 100× objective (UPLFLN 100×02 PH, oil immersion, NA=1.3). The pixel size corresponds to 70 nm. Cells and nucleoids were imaged, respectively, with phase contrast and DAPI channels. Images from random, non-overlapping fields were acquired. Pictures were processed uniformly using ImageJ and analizad using ObjectJ plugin.

Between 250–300 cells were measured for each sample, rendering coefficient of variation of no more than 15–20 % for length data and 20–30 % for area ones. Consequently, the standard error of the mean did not exceed 1 and 2 %, respectively. Error bars are not shown in the figures because their sizes are identical to the markers’. The lines are used to unite points from the same dataset.

## Results, discussion and conclusions

**Fig. 1.**
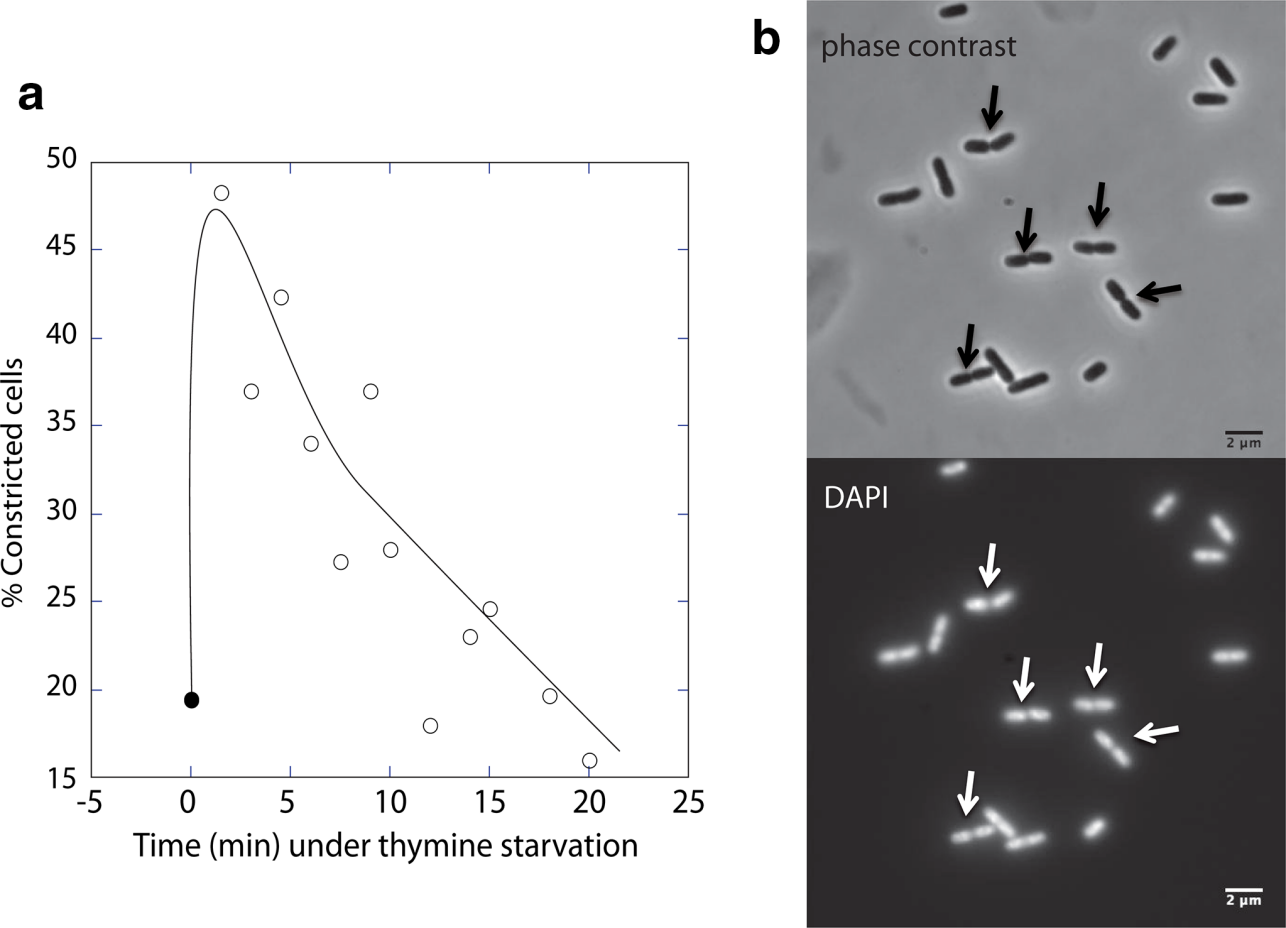
Constricted cells of strain MG1693 during early stages of thymine starvation. (a) Percentage. (b) Micrographs of phase contrast and DAPI-stained 5 min thymine starved cells. Arrows point to constrictions in cells with well-segregated nucleoids.

A striking doubling of percent constricted cells, from 20 % before deprivation of thymine to over 40 % within 5 min of treatment of *E. coli* MG1693 (Fig. 1a) supported the impression, gained from micrographs, that cells were shorter, whether by phase contrast or DAPI staining (Fig. 1b). This contrasts the long-term effects of thymine starvation, during which cells elongated hence their area increased by over sixfold. Some of these elongated cells appeared a-nucleated, and in others, the previously compacted nucleoids (usually located in mid-cell) dispersed and expanded to almost twice their initial value (data not shown).

**Fig. 2.**
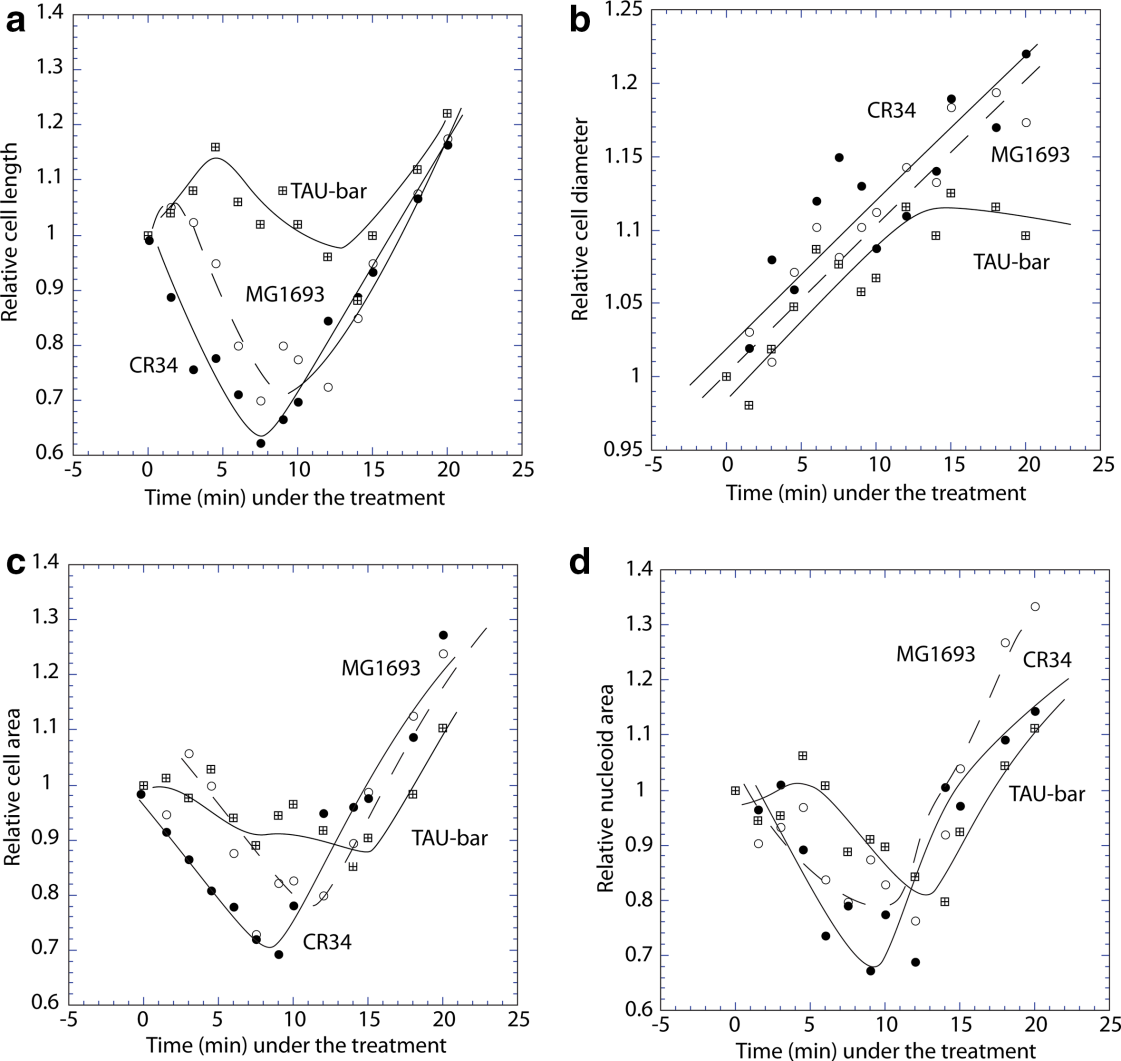
Short-term effects of thymine starvation on cell and nucleoid parameters in different *thyA* strains. Cell length (a) diameter (b) and area c), and nucleoid area (d) during 20 min in strain MG1693, CR34 and 15 TAU-bar.

The apparent remodelling of cell dimensions immediately after removal of thymine from the growth medium was quantified: significant drop (of 30–40 %) in cell length and area, and in nucleoid area, associated with 10–20 % width increase were indeed observed (Fig. 2a–d). Thus, *acceleration* of cell division and *remodelling* of cell dimensions occur during the first 10 min of the treatment. Increased number of c.f.u. during this immediate period, followed by the well-described loss of CFA supports this conclusion. The division-acceleration was confirmed by direct counting of cell concentrations in a Neubauer chamber (not shown). To test whether the enhanced divisions early during thymine deprivation is related to a peculiarity of strain K12 MG1693, the experiment was repeated with two other strains of *E. coli *, CR34 (K-12) and 15 TAU-bar, with similar responses (Fig. 2).

**Fig. 3.**
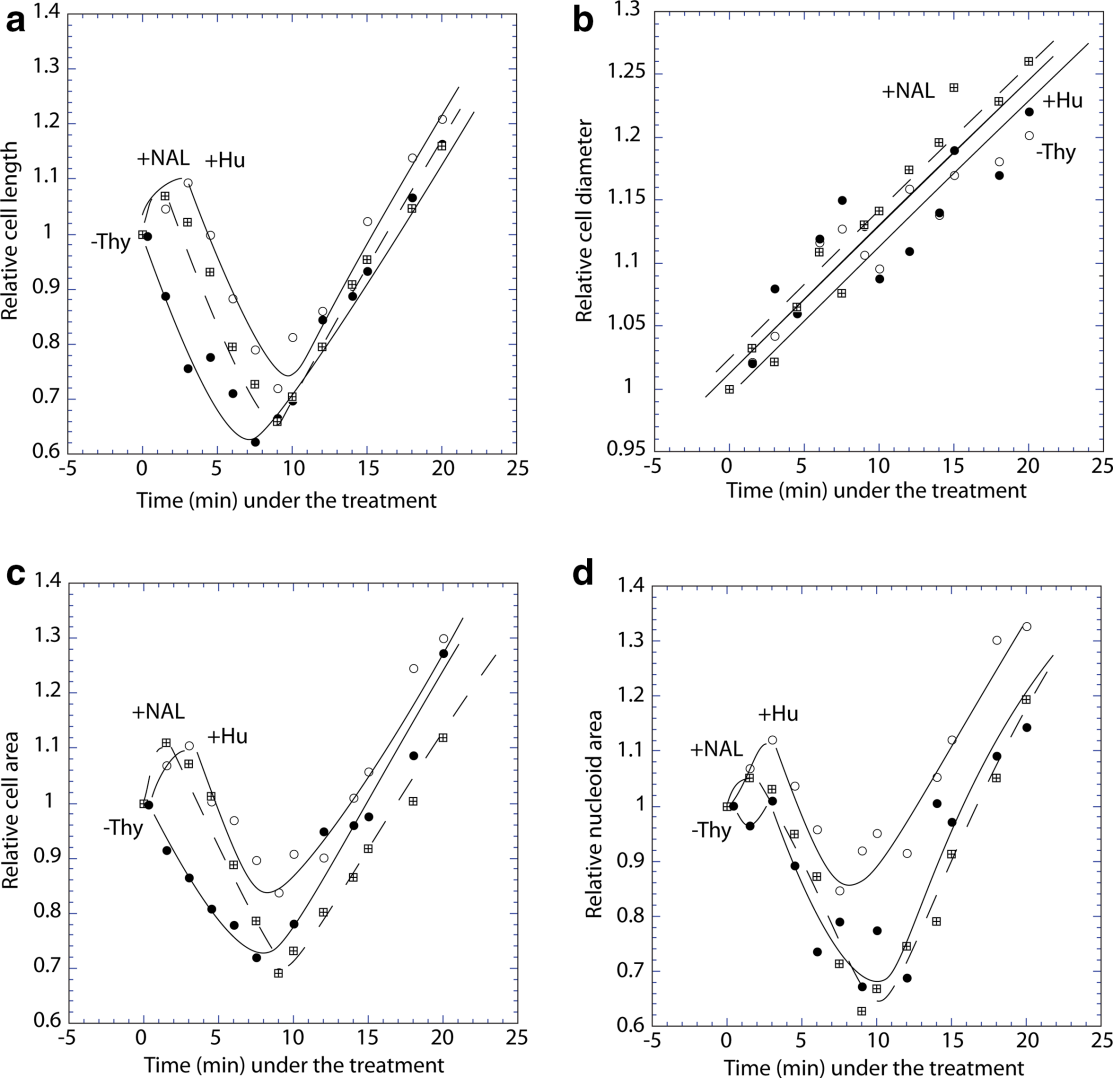
Cell and nucleoid parameters following block of DNA replication by various means in strain CR34: thymine starvation, HU addition and NAL addition: early effects on cell length (a) diameter (b) and surface area (c), and on nucleoid area (d).

These results demonstrate that thymine starvation enhances cell division before the following inhibition under long-term treatments. Using the same approach and experimental conditions, cell and nucleoid dimensions were analysed after blocking DNA replication by nalidixic acid or hydroxyurea (HU). Significant drops in both cell length and nucleoid area, associated with increased cell diameter were also observed here (Fig. 3), suggesting that enhanced cell division and remodelling of cell dimensions are general, perhaps universal, immediate reactions to arresting DNA replication in bacteria [6].

All measured parameters returned to values of untreated cells during one doubling time (~40 min) after restoring thymine (not shown), consistent with previous results demonstrating that the initiations inaugurated and aborted during the first 10 min of thymine starvation successfully resume and complete the ongoing round of replication upon replenishing thymine [7, 8].

The classical, *long-term* effects of DNA-replication inhibition on cell division and nucleoid morphology [1] apparently contradict the surprising, *immediate* changes affected by such treatment: significant drops in cell length and surface area, associated with a small rise in diameter and doubling in the proportion of constricted cells (Figs 1–3). Thus, inhibiting DNA replication enhances cell division in a certain proportion of cells before division-inhibition takes over at longer treatments.

**Fig. 4.**
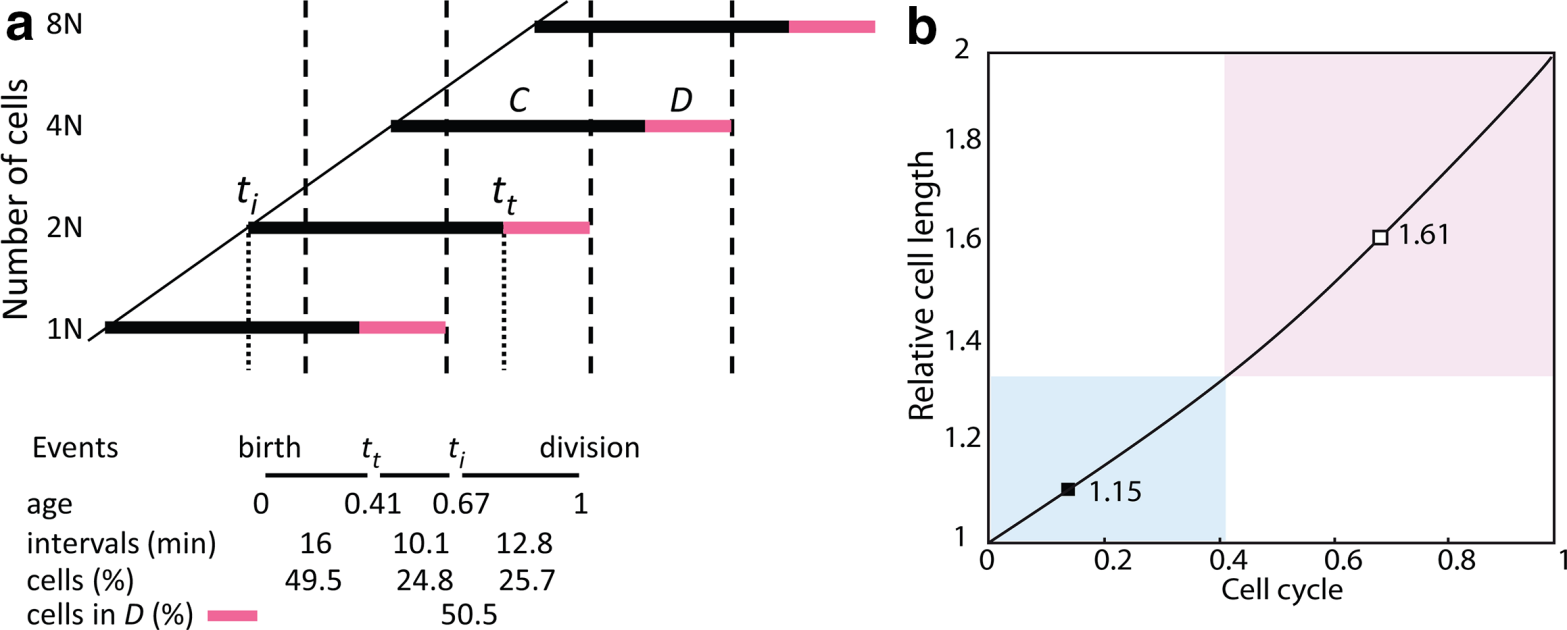
Schematic representation of the cell cycle in strain MG1693. Periods *C* and *D* are shown by solid and empty bars, respectively. The times of initiation and termination of replication round are designed as *t_i_* and *t_t_*, respectively. Percent cells for the intervals (0−*t_t_*), (*t_t_−t_i_*), (*t*_*i*_−1) and (*t_t_*−1) in an exponential, steady-state growing culture are calculated according to [19, 20] (a). Calculated average cell length for the intervals (0−*t_t_*) (blue square) and (*t_t_*−1) (pink rectangular) in exponential growth (b). The average length before *a_t_* (filled square) is obtained by ∑(n° cells×cell length in each period of 0.01 from 0 to *a_t_*)/∑(n° cells from 0 to *a_t_*) and after *a_t_* (empty square), by ∑(n° cells×cell length in each period of 0.01 from *a_t_* to 1)/∑(n° cells from *a_t_* to 1).

This study substantiates the coordinative relationships between DNA replication, nucleoid morphology, cell division and dimensions [9, 10]. It demonstrates that blocking DNA replication transiently enhances divisions in the population, likely of cells that had completed chromosome duplication, before fully inhibiting further divisions. To probe which cells perform advanced division according to the cell division cycle dogma [10–12], cells in the *D* period were assessed. Under the described growth conditions, where *τ*=39 min, *C*=68 min and *D*=23 min, about 0.505 of the cells are in the *D* period [12, 13] (Fig. 4a). Under these conditions, mean cell size is 1.391 [ = (0.495×1.152) + (0.505×1.618)] times the size of an average newborn cell (Fig. 4b). If all cells that are in the *D* period at onset of replication-arrest divide, average cell size becomes 0.917 [ = (0.328×1.152) + (0.671×0.805)] (Fig. 4b), corresponding to 0.66 of the mean of untreated cell size (0.917/1.391). This value is near the one obtained experimentally (Fig. 2a), consistent with the notion that all cells in the *D* period at onset of treatment do divide; they do not require further replication to divide, as was earlier proposed using a different approach [14]. FtsZ-ring assembly at mid-cell starts before replication-termination [15], consistent with the rapid increase of percent constricted cells observed during the first 5 min of thymine starvation (Fig. 1). Activation of FtsK, a divisome component which facilitates chromosome segregation during division [16, 17], can be envisioned as accelerating the movement of replicated chromosomes away from mid-cell and the FtsZ-ring, allowing the latter to constrict instantaneously. Thus, FtsK might be activated immediately upon replication block. Apparently, such an instantaneous activation of division has a metabolic nature rather than any gene expression. Therefore, alternatively, stimulation of cell division based on increased nucleotides and energy levels could accelerate the divisome and consequently shorten the *D* period upon arrest of replication.

Whatever the mechanism that enhances the division process here is, this physiological phenomenon is consistent with the finding that period *D* (time between replication-termination and subsequent cell division) is shortened upon extending *C* period (slowing the fork’s replication rate) by limiting thymine concentration in the growth medium of a *thyA* mutant of *E. coli* B/r [18].

At least two mechanisms seem to exist that couple DNA replication with cell division: the conventional one that inhibits division upon blocking uncompleted replication cycle and a new one, never observed before, to immediately accelerate divisions of cells that have terminated replication before onset of the treatment. This new mechanism acts *quickly* during *D* period; it could therefore be detected only by the sort of precise, highly frequent measurements as performed here. An alternative explanation may stem of the aborted initiations that had been observed during the first minutes of treatment [5], observations that led us to embark on this study. Any of these mechanisms may have evolved as a first defence to perturbations that affect survival: a cell that terminated a round of chromosome replication would better divide as quickly as possible before appearance of an anticipated catastrophe.
